# Pulmonary artery ablation to treat pulmonary arterial hypertension: a case report

**DOI:** 10.1186/s13256-015-0768-4

**Published:** 2015-12-16

**Authors:** Márcio Galindo Kiuchi, Bruno Rustum Andrea, Gustavo Ramalho da Silva, Sandro Barros Pinto Coelho, Luis Marcelo Rodrigues Paz, Shaojie Chen, Gladyston Luiz Lima Souto

**Affiliations:** 1 Department of Cardiac Surgery and Artificial Cardiac Stimulation, Sao Goncalo Hospital and Clinic, São Gonçalo, RJ Brazil; 2 Department of Medicine, Sao Goncalo Hospital and Clinic, São Gonçalo, RJ Brazil; 3 Electrophysiology Division, Department of Cardiology, Sao Goncalo Hospital and Clinic, São Gonçalo, RJ Brazil; 4 Interventional Cardiology Section, Department of Cardiology, Sao Goncalo Hospital and Clinic, São Gonçalo, RJ Brazil; 5 Department of Cardiology, Shanghai First People’s Hospital, Shanghai Jiao Tong University School of Medicine, Shanghai, China; 6 Department of Cardiology, Elisabethinen University Teaching Hospital Linz, Linz, Austria

**Keywords:** Pulmonary arterial hypertension, Catheter ablation, Denervation, 6-Minute walk test, Pulmonary arterial pressure

## Abstract

**Introduction:**

Idiopathic pulmonary arterial hypertension is defined as a group of diseases characterized by a progressive increase in pulmonary vascular resistance that results in right heart failure and premature death. Although therapies exist to improve hemodynamic instability and symptoms, there is no cure for pulmonary arterial hypertension and it remains a life-threatening condition. A recent study performed in China reported, for the first time, the effect of pulmonary arterial denervation on functional capacity and hemodynamics in patients with refractory idiopathic pulmonary arterial hypertension.

**Case presentation:**

We report a case of a 60-year-old white Brazilian man, with controlled hypertension and stage 2 obesity who complained of progressive fatigue with moderate to light exertion of approximately 1 year’s duration. During this period, he underwent myocardial perfusion scintigraphy without evidence of obstructive ischemic disease. He had no clinical evidence of systolic heart failure. He had undergone biological mitral valve replacement 3 years previously for mitral valve stenosis and ablation of atrioventricular nodal reentry tachycardia 18 months previously. At the time of valve replacement, he had no reported evidence of pulmonary arterial hypertension. His echocardiogram showed normal function of a mitral prosthesis, normal global left ventricular systolic function (left ventricular ejection fraction 62 % measured using the Teichholz method), stage I diastolic dysfunction, and a mean systolic pulmonary arterial blood pressure of 50 mmHg. In the 6-minute walk test, the patient walked 104 meters. Catheterization of his right heart chambers and pulmonary arteries confirmed the diagnosis of pulmonary hypertension. Electroanatomic reconstruction of the right ventricular outflow tract and pulmonary artery was performed under direct fluoroscopic visualization, and a merger was made with a formatted image of cardiac computed tomography angiography. Then we performed irrigated cardiac catheter ablation of the pulmonary trunk.

**Conclusions:**

At the patient’s 3-month follow-up, he showed improvement in functional class for fatigue on major exertion, increased distance walked in the 6-minute walk test, and reductions in pressure of both the right cavities and the pulmonary artery. Currently, with 6 months of clinical follow-up, the patient has maintained his functional classification and is pedaling his bicycle.

## Introduction

Idiopathic pulmonary arterial hypertension is defined as a group of diseases characterized by a progressive increase in pulmonary vascular resistance that results in right heart failure and premature death [1–7]. Recent therapeutic advances have improved the treatment options, including prostanoids, endothelin receptor antagonists, and phosphodiesterase type 5 inhibitors [3–6, 8, 9]. The pathogenesis of idiopathic pulmonary arterial hypertension is believed to be due to an imbalance between locally produced vasodilators and vasoconstrictors [10]. Recent studies have demonstrated that vascular wall remodeling also contributes to elevated pulmonary vascular resistance [11]. A meta-analysis of 23 randomized controlled trials demonstrated a reduction in mortality of patients who used targeted therapies approved for use in patients with pulmonary arterial hypertension [10].

## Case presentation

A 60-year-old white Brazilian man, with controlled hypertension and stage 2 obesity presented to our institution with a complaint of progressive fatigue with moderate to light exertion of approximately 1 year’s duration. During that period, he had undergone myocardial perfusion scintigraphy without evidence of obstructive ischemic disease. He had no clinical evidence of systolic heart failure. He had undergone biological mitral valve replacement 3 years previously for mitral valve stenosis and had undergone ablation of atrioventricular nodal reentry tachycardia 18 months previously. At the time of valve replacement, there was no reported evidence of pulmonary arterial hypertension. The patient’s medication list included aspirin 100 mg/day, carvedilol 50 mg/day, atorvastatin 10 mg/day, and losartan 25 mg/day. His echocardiogram showed normal function of a mitral prosthesis, global left ventricular systolic function within normal limits (left ventricular ejection fraction 62 % measured using the Teichholz method), stage I diastolic dysfunction, and mean pulmonary arterial systolic blood pressure of 50 mmHg. In the 6-minute walk test, the patient walked 104 meters (Table 1). Catheterization of his right heart chambers and pulmonary arteries confirmed the diagnosis of pulmonary hypertension (Table 2). During the follow-up period, therapy with nifedipine and sildenafil was not tolerated secondary to orthostatic hypotension. The patient was referred for radiofrequency ablation of the pulmonary artery trunk for the treatment of refractory pulmonary hypertension.

**Table 1:** 6-Minute walk test results

Time point	Distance
Baseline	104 m
3 months after ablation	250 m
6 months after ablation	302 m

**Table 2:** Right catheterization and mean systolic blood pressure measurements before and after pulmonary artery ablation

Structures	Baseline	3 months	6 months
Right pulmonary arterial pressure, mmHg	46	35	31
Left pulmonary arterial pressure, mmHg	46	33	30
Pulmonary artery trunk pressure, mmHg	47	36	29
Right ventricular pressure, mmHg	40	33	30
Right atrial pressure, mmHg	20	14	11

**Fig. 1:**
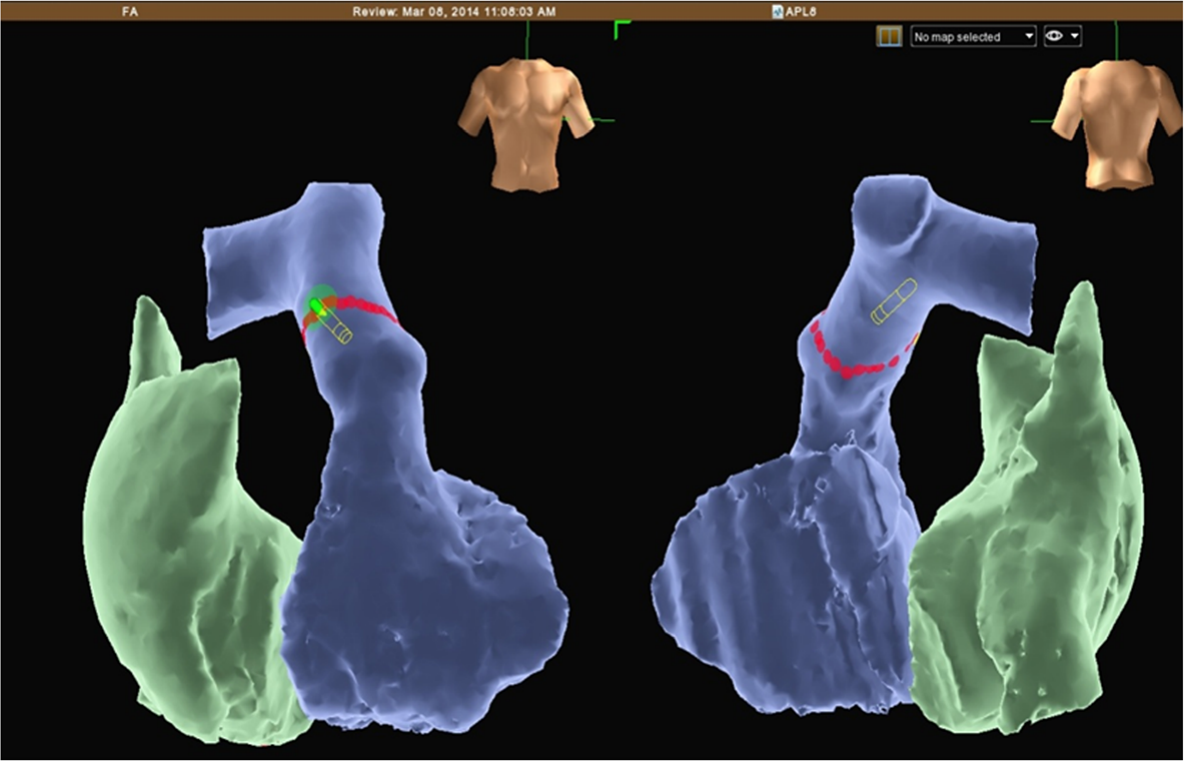
Electroanatomic reconstruction of the right ventricular outflow tract and pulmonary artery was performed using the EnSite Velocity Cardiac Mapping System, and a merger was made with a formatted image obtained by performing cardiac computed tomography angiography

The procedure was performed in the catheterization laboratory with direct visualization using fluoroscopy and radiopaque contrast dye. The patient remained under unconscious sedation. Catheterization of the right femoral artery via the standard Seldinger technique was performed using an 8-French valved short sheath after subcutaneous injection of a local anesthetic. Subsequently, this sheath was replaced with a steerable long sheath (Agilis®; St. Jude Medical, St. Paul, MN, USA) using the standard over-the-wire technique. Unfractionated heparin was administered intravenously, targeting an activated coagulation time between 250 and 350 seconds. Electroanatomic reconstruction of both the right ventricular outflow tract and pulmonary artery was performed using the EnSite Velocity Cardiac Mapping System (St. Jude Medical) under direct fluoroscopic visualization, and a merger was made with the formatted image obtained by performing cardiac computed tomography angiography (Fig. 1). The Agilis® sheath was advanced into the right ventricular outflow tract just before reaching the pulmonary valve. Through this long sheath, we introduced an ablation catheter with an open irrigated tip (St. Jude Medical). The parameters used for each application according to our protocol were as follows: power of 5 W, maximum temperature of 48 °C, 60-second duration in each spot, maximum impedance variation of 10 % from baseline values, and an irrigation flow rate of 17 ml/minute, which created a circle in the pulmonary artery trunk.

The patient was discharged the next morning. No noteworthy changes before or after the procedure or before discharge in the patient’s radiographic or echocardiographic laboratory parameters were seen. The patient’s blood pressure in both the right heart chambers and the pulmonary artery were determined using catheterization before and at 3 and 6 months after the procedure. The results are shown in Table 2. At the patient’s 3-month follow-up examination, he showed an improvement in functional class for fatigue with major exertion. He also demonstrated an increased distance walked in the 6-minute walk test and reduction of the pressures in both the right cavities and the pulmonary artery. Currently, with 6 months of clinical follow-up, he has maintained his improvement in functional classification and is pedaling his bicycle.

## Discussion

There is no cure for pulmonary arterial hypertension, and it remains a life-threatening disorder, though therapies can improve patients’ symptoms, hemodynamics, and outcomes [12]. A recent study performed in China reported, for the first time, the effect of pulmonary arterial denervation on functional capacity and hemodynamics in patients with refractory idiopathic pulmonary arterial hypertension [13].

## Conclusion

The ablation of the pulmonary artery could be a promising tool in the future for the treatment of pulmonary hypertension.

## Consent

Written informed consent was obtained from the patient for publication of this case report and any accompanying images. A copy of the written consent is available for review by the Editor-in-Chief of this journal. The ethics committee (Paola Baars Gomes Moises, Luis Marcelo Rodrigues Paz, Humberto Cesar Tinoco, and Jonny Shogo Takahashi) at our institution approved the execution of the case.
